# Locoregional Failure During and After Short-course Radiotherapy Followed by Chemotherapy and Surgery Compared With Long-course Chemoradiotherapy and Surgery: A 5-Year Follow-up of the RAPIDO Trial

**DOI:** 10.1097/SLA.0000000000005799

**Published:** 2023-01-20

**Authors:** Esmée A. Dijkstra, Per J. Nilsson, Geke A.P. Hospers, Renu R. Bahadoer, Elma Meershoek-Klein Kranenbarg, Annet G.H. Roodvoets, Hein Putter, Åke Berglund, Andrés Cervantes, Rogier M.P.H. Crolla, Mathijs P. Hendriks, Jaume Capdevila, Ibrahim Edhemovic, Corrie A.M. Marijnen, Cornelis J.H. van de Velde, Bengt Glimelius, Boudewijn van Etten

**Affiliations:** * Department of Medical Oncology, University Medical Center Groningen, University of Groningen, Groningen, The Netherlands; † Department of Surgery, Karolinska University Hospital, Stockholm, Sweden; ‡ Department of Surgery, Leiden University Medical Center, Leiden, The Netherlands; § Department of Medical Statistics and Bioinformatics, Leiden University Medical Center, Leiden, The Netherlands; ∥ Department of Immunology, Genetics and Pathology, Uppsala University, Uppsala, Sweden; ¶ Department of medical oncology, Biomedical Research Institute Incliva, University of Valencia, Valencia, Spain; # Department of Surgery, Amphia Hospital, Breda, The Netherlands; ** Department of Medical Oncology, Northwest Clinics, Alkmaar, The Netherlands; †† Department of Medical Oncology, Vall Hebron Institute of Oncology (VHIO), Vall Hebron University Hospital, Autonomous University of Barcelona (UAB), Barcelona, Spain; ‡‡ Department of surgical oncology, Institute of Oncology Ljubljana, Ljubljana, Slovenia; §§ Department of Radiation Oncology, Netherlands Cancer Institute, Amsterdam, The Netherlands; ∥∥ Department of Radiation Oncology, Leiden University Medical Center, Leiden, The Netherlands; ¶¶ Department of Surgery, University Medical Center Groningen, University of Groningen, Groningen, The Netherlands

**Keywords:** locally advanced rectal cancer, locoregional failure, locoregional recurrence, total neoadjuvant treatment

## Abstract

**Objective:**

To analyze risk and patterns of locoregional failure (LRF) in patients of the RAPIDO trial at 5 years.

**Background:**

Multimodality treatment improves local control in rectal cancer. Total neoadjuvant treatment (TNT) aims to improve systemic control while local control is maintained. At 3 years, LRF rate was comparable between TNT and chemoradiotherapy in the RAPIDO trial.

**Methods:**

A total of 920 patients were randomized between an experimental (EXP, short-course radiotherapy, chemotherapy, and surgery) and a standard-care group (STD, chemoradiotherapy, surgery, and optional postoperative chemotherapy). LRFs, including early LRF (no resection except for organ preservation/R2 resection) and locoregional recurrence (LRR) after an R0/R1 resection, were analyzed.

**Results:**

Totally, 460 EXP and 446 STD patients were eligible. At 5.6 years (median follow-up), LRF was detected in 54/460 (12%) and 36/446 (8%) patients in the EXP and STD groups, respectively (*P*=0.07), in which EXP patients were more often treated with 3-dimensional-conformed radiotherapy (*P*=0.029). In the EXP group, LRR was detected more often [44/431 (10%) vs. 26/428 (6%); *P*=0.027], with more often a breached mesorectum (9/44 (21%) vs. 1/26 (4); *P*=0.048). The EXP treatment, enlarged lateral lymph nodes, positive circumferential resection margin, tumor deposits, and node positivity at pathology were the significant predictors for developing LRR. Location of the LRRs was similar between groups. Overall survival after LRF was comparable [hazard ratio: 0.76 (95% CI, 0.46–1.26); *P*=0.29].

**Conclusions:**

The EXP treatment was associated with an increased risk of LRR, whereas the reduction in disease-related treatment failure and distant metastases remained after 5 years. Further refinement of the TNT in rectal cancer is mandated.

Over the past decades, improved imaging, preoperative radiotherapy (RT) or conformal radiotherapy (CRT), and total mesorectal excision (TME) surgery have resulted in improved local control rates in patients with rectal cancer.^1–3^ Despite these improvements, the systemic relapse rate has remained largely unaltered. The concept of total neoadjuvant treatment (TNT) was introduced to address the distant metastasis (DM) rate. Recently, the results of the RAPIDO trial demonstrated that preoperative short-course radiotherapy (scRT) followed by systemic chemotherapy (ie, TNT) resulted in a decreased disease-related treatment failure (DrTF) rate (mainly by a decrease in DM compared with standard CRT at 3 years of follow-up in high-risk locally advanced rectal cancer).^4^ However, less is known about locoregional failure (LRF) rates after TNT.

LRF can occur at different time points during rectal cancer management using TNT. In poor or nonresponders to the neoadjuvant treatment, the tumor may be irresectable or lead to an R2 resection causing an early LRF (eLRF). In patients who undergo an R0 or R1 resection, an LRF may occur during follow-up as an locoregional recurrence (LRR).

The aim was to investigate the rate and describe patterns of LRFs, including LRRs, in the experimental (EXP) and the standard-care (STD) treatment groups in the RAPIDO trial. Moreover, survival after an LRF was analyzed.

## MATERIALS AND METHODS

### Patient Selection

The RAPIDO trial is an international, multicenter, phase III, randomized trial. It was approved by the institutional review boards of participating institutions (2010-023957-12). Details of the trial have been reported.^5^ In short, patients with rectal adenocarcinoma, less than 16 cm from the anal verge at endoscopy and with high-risk features on magnetic resonance imaging (MRI) [cT4a/b, cN2, enlarged lateral lymph nodes (ELLNs) considered to be metastatic, extramural vascular invasion (EMVI+) or involved mesorectal fascia (MRF+)] were randomized (1:1) to EXP or STD treatment. Patients were included between 2011 and 2016. The data lock for this report was March 11, 2022.

### Treatments

The EXP treatment consisted of 5×5 Gy RT, followed by 6 cycles of CAPOX or 9 cycles of FOLFOX4. Within 2 to 4 weeks after this treatment, TME surgery was performed. The STD treatment consisted of long-course RT (28–25×1.8–2.0 Gy) and concurrent capecitabine, followed by surgery after 8±2 weeks. According to hospital policy, patients in the STD group could receive postoperative 8 cycles of CAPOX or 12 cycles of FOLFOX4. RT target volumes did not differ between the EXP and STD groups. The results from the primary and some secondary endpoints of the RAPIDO trial have been reported.^6,7^

Restaging was performed in the EXP group 1 to 2 weeks after the last chemotherapy cycle and 2 to 3 weeks before planned surgery in the STD group. Restaging was performed by computed tomography (CT) of the thorax, abdomen, and pelvis and MRI of the pelvis. In the EXP group, an additional MRI of the pelvis was recommended in the middle of the neoadjuvant chemotherapy (week 12–14) to disclose any signs of progression. Treatment response was assessed after neoadjuvant treatment (based on baseline and restaging MRI reports) and after surgery (based on pathology reports). For this report, all patients with a decrease in T-stage and/or N-stage compared with baseline MRI stage were defined as good responders (ie, downstaging was accomplished).

### Follow-up

Follow-up was according to a standardized protocol. Outpatient visits were scheduled at 6, 12, 24, 36, and 60 months after surgery. The study protocol mandated a CT scan of the thorax and abdomen (or chest x-ray and liver ultrasound) at 12 and 36 months after surgery as a minimum. On indication, other diagnostics were performed to confirm or detect recurrent disease.

### Outcomes

A secondary endpoint in the RAPIDO trial and the primary endpoint in this study was LRF, including eLRF and LRR. eLRF was defined as patients having no surgery/nonresectional surgery unless this was in an organ preservation setting or R2 resection. Patients who were lost to follow-up, withdrew informed consent, or died before surgery were excluded from analyses.

**FIGURE 1:**
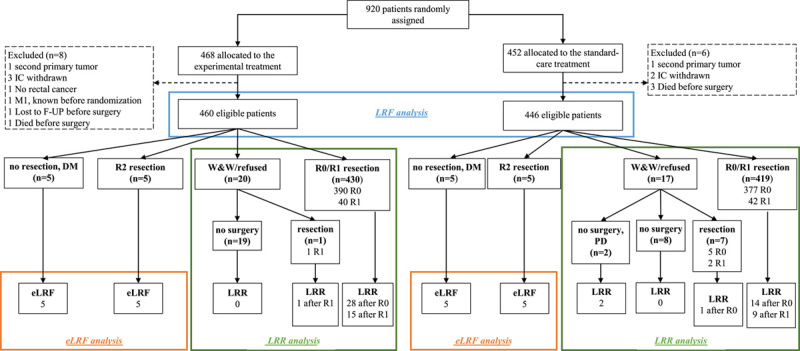
Consort diagram. Patients entering a W&W program or who “refused surgery” according to the case record forms were grouped together since the predominant reason for the refusers was no remaining tumor/no need for surgery. These patients were included in the LRR analysis. The 2 patients who initially entered a W&W strategy/refused surgery but later developed regrowth without having surgery were scored as LRR. When W&W patients with tumor regrowth underwent a curative resection, this was not scored as LRR. However, when regrowth was subsequent to a radical resection in W&W patients, this was scored as LRR. F-UP indicates follow-up; IC, informed consent; PD progressive disease.

An LRR was defined as a locoregionally recurrent disease after a previous R0 or R1 resection. When watch-and-wait (W&W) patients with tumor regrowth underwent a curative resection, this was not scored as LRR. However, any subsequent local recurrence after a radical resection in W&W patients was considered as an LRR. Patients refusing surgery were grouped with those entering the W&W strategy, as the predominant reason for refusal was no residual tumor. The 2 patients who were not operated up-front and much later had locally progressive disease were scored as LRR (Fig. 1 and Table S1, Supplemental Digital Content 1, http://links.lww.com/SLA/E421). Histopathological confirmation of an LRR was not mandatory when indicated by CT, MRI, and/or positron emission tomography scans. Secondary outcomes included the location of the LRRs and the treatment of LRF. For this report, updated results for the RAPIDO endpoints DrTF, DM, and overall survival (OS) at 5 years were analyzed.

### Location of LRR

The location of recurrent disease was recorded in the CRFs and centrally reviewed by imaging reports (MRI, CT, and positron emission tomography) and/or histology reports. Locations were classified according to Kusters et al.^8^ (Supplementary Appendix p. 6)

In patients with large or multifocal LRRs, all involved subsites were recorded.

### Statistics

The RAPIDO trial was powered for the primary endpoint (DrTF) but not for any secondary endpoints, including LRF reported here. LRF analyses were performed on an intention-to-treat basis on all eligible patients. LRR analyses were performed in all eligible patients who underwent an R0 or R1 resection (and in 2 nonoperated patients who later developed progressive disease). Proportions were compared with χ^2^ tests and continuous parameters, depending on the distribution of the data, with the *t* test or Mann-Whitney *U* test. When a patient developed DM within 3 months (before or after) of an LRF, the DM was defined as synchronous. Univariate and multivariate Cox regression analyses were used to calculate the influence of baseline characteristics on the occurrence of LRF and LRR and to calculate the influence of surgical and histopathological characteristics on LRR. The median follow-up was calculated by the reversed Kaplan-Meier method. The median survival time after the diagnosis of LRF was calculated by the Kaplan-Meier method. Differences were assessed using the log-rank test. Cumulative incidence of DrTF, DM, and OS were calculated accounting for all causes of death as a competing risk. For all competing risk analyses, hazard ratios (HRs) and 95% CI were calculated by Cox regression. In univariate analyses, a *P* value of ≤0.10, and in all other statistical analyses *P*≤0.05 was considered statistically significant. SPSS for Windows (version 28, SPSS, Chicago, IL) and R-studio were used for the statistical analyses.

## RESULTS

### Study Population

Nine hundred and twenty patients were randomized in the RAPIDO trial, of whom 906 (460 in the EXP and 446 patients in the STD group) were eligible for the LRF analyses (Fig. 1). Patients who underwent an R0/R1 resection, 857/906, were included in the LRR analysis (431 in the EXP and 426 in the STD group). The median follow-up was 5.6 years (interquartile range: 5.4–7.5).

### Overall LRF

**FIGURE 2:**
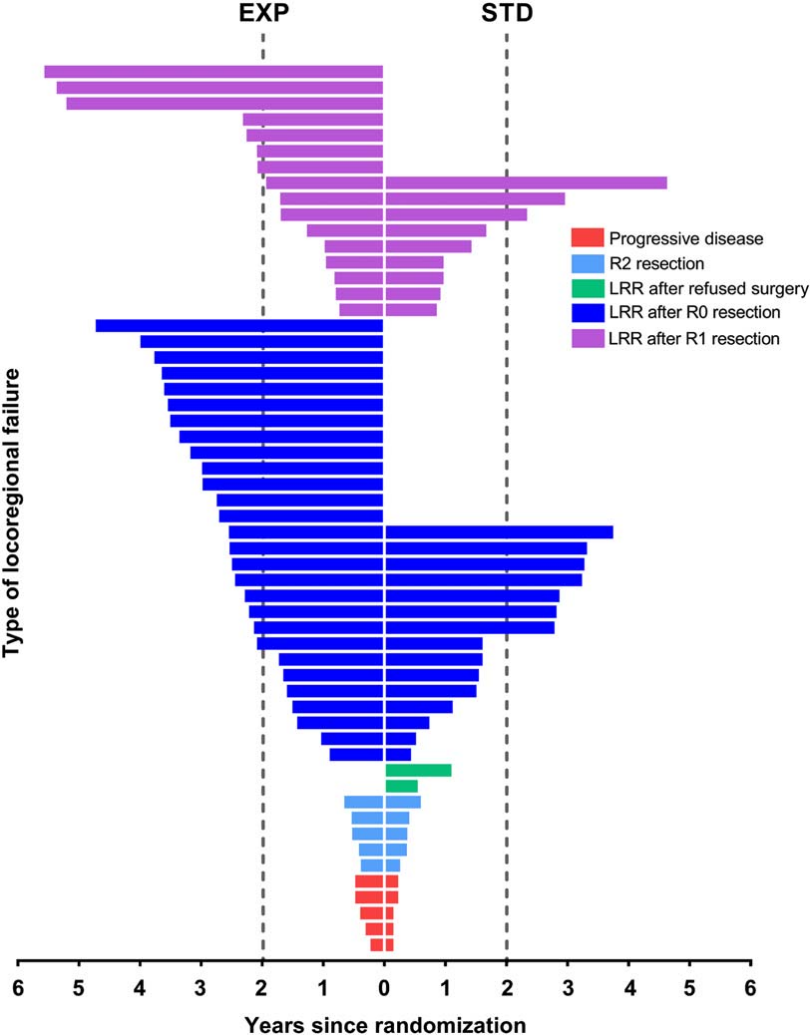
Plot development of locoregional failure against time in years after randomization. Red: no resection surgery for other reasons than entering a W&W strategy (5 vs. 5). Light blue: R2 (residual tumor locally (all these patients also had distant metastases) (5 vs. 5). Green: locoregionally progressive disease after having refused surgery (0 vs. 2, referred to the LRR group) Dark blue: LRR after an R0 resection (28 vs. 15). Pink: LRR after an R1 resection (16 vs. 9).

An LRF was detected in 54/460 (11.7%) and 36/446 (8.1%) patients in the EXP and STD groups, respectively (*P*=0.07). Baseline characteristics of patients included in the LRF analyses and in whom an LRF was detected are provided in Table S1, Supplemental Digital Content 1, http://links.lww.com/SLA/E421. No significant differences in baseline high-risk criteria between the 2 groups were found. Patients in the EXP group with an LRF received more often 3-dimensional-conformed radiotherapy (3D-CRT) compared with those in the STD group (*P*=0.029). The different types of LRFs, in relation to time after randomization, are demonstrated in Fig. 2.

### eLRF

An eLRF occurred in 20 patients (10/460 in the EXP and 10/446 in the STD group, Table S2, Supplemental Digital Content 1, http://links.lww.com/SLA/E421). Eight and 10 of these patients also developed DM in the EXP and STD groups, respectively. All of them developed DM before or synchronously with the eLRF. In univariate analyses, distance from the anal verge (*P*=0.049), presence of ELLNs (*P*=0.002), and EMVI+ (*P*=0.014) were associated with an eLRF, but no statistically significant associations were found in the multivariate analysis (Table S3, Supplemental Digital Content 1, http://links.lww.com/SLA/E421).

### LRR After an R0 or R1 Resection

Totally, 886/912 (97%) patients were included in the LRR analyses (Fig. 1). Of them, 857 (97%) underwent an R0 or R1 resection. There were no statistically significant differences in R0 or R1 resection rates between the EXP and STD groups. A higher rate of LRR was detected in the EXP group compared with the STD group; 44/431 (10.2%) and 26/428 (6.1%), *P*=0.027. Following an R0 resection, LRR was more often detected in the EXP group (7.2% vs. 3.9%; *P*=0.049), and a similar numerical difference, but not statistically significant, was observed for R1 resected patients (39.0% vs. 20.5%; *P*=0.06).

Except for the mesorectum being more often breached in the EXP group, Table S4, Supplemental Digital Content 1, http://links.lww.com/SLA/E421 demonstrates no statistically significant differences in high-risk criteria and histopathological characteristics between LRR patients of the EXP and STD groups. In a multivariate Cox regression analysis (Table 1), the EXP treatment (*P*=0.014) and ELLNs (*P*=0.042) were associated with LRR.

**TABLE 1:** Univariate and Multivariate Cox Regression Analyses for Locoregional Recurrence Regarding Allocation Group, Distance From the Anal Verge and High-risk Factors at Baseline in Patients Who Underwent an R0 or an R1 Resection

		Univariate analyses			Multivariate analyses		
Variable	Category	n	HR (95% CI)	*P*	n	HR (95% CI)	*P*
Treatment	Standard-care	**426**	**1**	—	**426**	**1**	—
	Experimental	**431**	**1.84 (1.12–3.02)**	**0.017**	**431**	**1.87 (1.14–3.07)**	**0.014**
Distance from anal verge (endoscopy)^*^	<5 cm	196	1	0.829	—	—	—
	5–10 cm	318	1.19 (0.63–2.27)	—	—	—	—
	≥10 cm	282	1.094 (0.53–2.04)	—	—	—	—
Clinical T4	No	582	1	—	—	—	—
	Yes	275	0.99 (0.59–1.65)	0.959	—	—	—
Clinical N2	No	267	1	—	—	—	—
	Yes	590	1.38 (0.80–2.39)	0.252	—	—	—
Clinical ELLN	No	**723**	**1**	—	**723**	**1**	—
	Yes	**134**	**1.74 (0.99–3.04)**	**0.053**	**134**	**1.79 (1.02–3.13)**	**0.042**
Clinical EMVI+	No	557	1	—	—	—	—
	Yes	300	1.13 (0.69–1.85)	0.621	—	—	—
Clinical MRF+	No	271	1	—	—	—	—
	Yes	586	1.25 (0.74–2.12)	0.412	—	—	—

Bold values indicate statistical significance *p* ≤ 0.05.

Test for interaction is *P*=0.89.

* In 61 patients, the distance from the anal verge was unknown.

MRF indicates mesorectal fascia.

The time from surgery to the detection of an LRR was 1.8 years (interquartile range: 1.2–2.6) in the EXP and 1.2 years (0.6–2.7) in the STD group (*P*=0.31), respectively. When an LRR was detected, 36/70 (52%) had prior or synchronous DM, being similar in both groups (EXP 22/44 (50%) vs. STD 14/26 (54%) (*P*=0.84).

Regarding radiation technique, patients from the EXP group developed more often an LRR after 3D-CRT compared with the STD group (11.6% (37/320) vs. 6.0% (18/298); *P*=0.016). The LRR rate was comparable after intensity-modulated radiation therapy (IMRT)/volumetric-modulated arc therapy (VMAT) (6.3% (7/111) vs. 6.2% (8/130) in the EXP and STD groups, respectively; *P*=0.96). Overall, a comparable number of patients developed an LRR after a (low) anterior resection or an abdominoperineal resection (8% vs. 7%). After Hartmann’s procedure (n=37), an LRR was detected in 11 (30%) patients. Regarding TME quality, an intraoperative breach of the mesorectum occurred more often in the EXP group compared with the STD group (11% (42/378) vs. 6% (25/389); *P*=0.022). In patients with a breached mesorectum, LRR was more often detected in the EXP group (21% (9/42) vs. 4% (1/25); *P*=0.053). In the Cox regression analyses on histopathological factors, the EXP treatment (*P*=0.004), positive circumferential resection margin (*P*<0.0001), tumor deposits (*P*=0.004), and ypN-stage (*P*=0.014) were associated with an LRR (Table 2).

**TABLE 2:** Univariate and Multivariate Cox Regression Analyses of Locoregional Recurrence Regarding Allocation Group and Pathologic Factors After Surgery in Patients Who Underwent an R0/R1 Resection

		Univariate analyses			Multivariate analyses		
Variable	Category	n	HR (95% CI)	*P*		HR (95% CI)	*P*
Treatment	Standard-care	**426**	**1**	—	**287**	**1**	**0.004**
	Experimental	**431**	**1.87 (1.14–3.07)**	**0.014**	**234**	**2.38 (1.33–4.27)**	—
CRM	CRM−	**777**	**1**	—	**455**	**1**	**<0.0001**
	CRM+	**80**	**7.18 (4.36–11.82)**	**<0.0001**	**66**	**4.13 (2.25–7.59)**	—
Differentiation grade	Well	**151**	**1**	**0.016**	138	1	0.631
At pathology^*^	Moderate	**377**	**0.74 (0.40–1.35)**	—	314	0.85 (0.44–1.65)	—
	Poor	**82**	**1.87 (0.91–3.83)**	—	69	1.21 (0.54–2.73)	—
Mesorectum	Intact	**700**	**1**	—	471	1	0.800
Assessment^*^	Breached	**67**	**2.37 (1.21–4.68)**	**0.012**	50	1.11 (0.50–2.48)	—
EMVI at pathology^*^	EMVI−	**744**	**1**	—	443	1	0.896
	EMVI+	**105**	**4.22 (2.53–7.01)**	**<0.0001**	78	1.05 (0.54–2.02)	—
Tumor deposits^*^	No	**749**	**1**	—	**445**	**1**	**0.004**
	Yes	**95**	**3.96 (2.34–6.70)**	**<0.0001**	**76**	**2.43 (1.32–4.48)**	—
ypN-stage^*^	ypN0	**604**	**1**	**<0.0001**	**336**	**1**	**0.014**
	ypN1	**166**	**3.03 (1.72–5.33)**	—	**115**	**2.19 (1.09–4.41)**	—
	ypN2	**79**	**5.82 (3.18–10.64)**	—	**70**	**2.97 (1.38–6.38)**	—
Tumor size at	<40 mm	**703**	**1**	**0.001**	410	1	0.075
pathology^*^	≥40 mm	**137**	**2.41 (1.43–4.06)**		111	1.76 (0.94–3.29)	—

Bold values indicate statistical significance *p* ≤ 0.05.

Two patients did not undergo curative surgery; therefore, the initial number of patients included is 857 instead of 859.

*CRM* indicates circumferential resection margin.

* In case the variable was unknown for a patient, the patient was set to missing. Therefore, the number of patients included in the multivariate analysis is considerably lower.

Post-treatment restaging MRI data was available for 841/859 (97.9%) patients. In total, 632/841 (75.1%) patients were assessed as good responders (80.1% vs. 70.1% (*P*<0.0001) in the EXP and STD groups, respectively. Overall, recurrent disease was less often detected in MRI-based good responders (6.8% vs. 12.0%; *P*=0.020). On the basis of histopathology reports, 773/857 (90.2%) were assessed as good responders (93.0% vs. 87.3% (*P*=0.008) in the EXP and STD groups, respectively). As with the MRI-based response evaluation, an LRR was significantly less often detected in good responders (6.9% vs. 16.9%; *P*<0.0001).

Table S5, Supplemental Digital Content 1, http://links.lww.com/SLA/E421 provides the location(s) of the 44 and 26 LRRs of the EXP and STD groups, respectively. No statistically significant differences between the EXP and STD groups concerning location and number of involved locations were observed. However, presacral (19 vs. 9 patients) and anastomotic (14 vs. 3 patients). LRRs occurred numerically more often in the EXP compared with the STD group.

### Treatment of LRF

The treatment intention (curative/palliative) for patients with an LRF did not differ between the 2 groups (*P*=0.48). All 20 patients with an eLRF were treated with palliative intent. In case of an LRR, reirradiation was delivered to 11/44 (25%) and 1/26 (4%) of the patients of the EXP and STD groups, respectively. Among these reirradiated patients, 7 in the EXP group and 1 in the STD group underwent surgery. Two patients in the EXP and 4 patients in the STD group received only best supportive care for their LRR. Overall, surgical resection of the LRR was performed in 22/44 (50%) patients in the EXP group and 11/26 (42%) patients in the STD group. In both groups, when surgery was performed, it was mostly with curative intent (82%). The median survival of patients with an LRF was 1.6 years (0.6–3.2) in the EXP group and 1.2 years (0.4–2.4) in the STD group (*P*=0.29) (Figure S1).

### Five-year Update of Oncological Outcomes of the RAPIDO Trial

At 5 years, the cumulative probability of DrTF was 27.8% (95% CI, 23.7–31.8) in the EXP group and 34.0% (95% CI, 29.6–38.4) in the STD group (HR: 0.79 (95% CI, 0.63–1.00); *P*=0.0480). The cumulative probability of DM at 5 years in the EXP group was 23.0% (95% CI, 19.2–26.8) and 30.4% (95% CI, 26.1–34.7) in the STD group (HR: 0.73 (95% CI, 0.57–0.93); *P*=0.011). At 5 years, the cumulative probability of OS was 81.7% (95% CI, 78.2–85.22) in the EXP group compared with 80.2% (95% CI, 76.5–83.9) in the STD group (HR: 0.91 (95% CI, 0.70–1.19); *P*=0.50).

## DISCUSSION

The results from the RAPIDO trial demonstrated that the EXP treatment is associated with a decreased incidence of DM and an increased rate of pathological complete response (pCR), and comparable LRF rates to the STD treatment at 3 years of follow-up.^4^ With a longer follow-up (median 5.6 y), the rates of both LRF and LRR are higher in the EXP group compared with the STD group (12 vs. 8%, *P*=0.07 and 10 vs. 6%, *P*=0.03). Thus, although the RAPIDO trial demonstrated favorable outcomes concerning systemic control with a TNT approach, this report indicates a risk of compromising local control with the EXP treatment, despite a doubled chance to obtain pCR. With the prolonged follow-up, OS remains similar between the EXP and STD groups.

eLRF are rarely seen but occur at similar rates in the EXP and the STD groups of the RAPIDO trial. Most patients with eLRF, both among EXP and STD, also developed DM before or in conjunction with the eLRF. Patients with eLRF seem to represent a subset of patients who have an extremely poor prognosis irrespective of the treatment approach. We demonstrated that the vast majority of eLRF patients had cN2 and mesorectal fascia involvement. Hopefully, future research may result in the identification of these patients pretherapeutically (eg, via biomarkers) and offer more personalized approaches. The rareness of eLRF constitutes an obstacle to meaningful statistical analyses of risk factors.

Of the patients in the RAPIDO trial, the overall LRR rate is 7.8% which is comparable to literature considering the locally advanced stages included.^2,3,9,10^ However, the statistically significantly increased LRR rate in the EXP group compared with STD raises several questions. The analyses of patient and tumor characteristics reveal no imbalances between the 2 groups. However, a breach of the mesorectum is associated with an LRR.^11^ A mesorectal breach occurred more often in the EXP group, and the increased risk of LRR in the EXP group was most pronounced in the breached group. scRT per se (compared with primary surgery) did not affect the plane of surgery in the MRC-CR07 trial,^12^ but it may be speculated that the prolonged preoperative chemotherapy in the EXP group could yield a more fragile or fibrotic mesorectum and poorer specimen quality. This may, thus, provide 1 possible explanation for the increased rate of LRR in the EXP group.

The radiation technique was the only statistically significant different baseline/initial treatment characteristic when comparing the 2 groups of LRF patients. Patients from the EXP group received more often 3D-CRT, whereas the STD group received IMRT/VMAT to a higher degree. During the time of RAPIDO inclusion (2011-2016), IMRT/VMAT, a relatively new radiation technique at the time, had become standard-of-care more commonly in patients treated with long-course CRT compared with scRT, although this difference did not reach statistical significance. There is no obvious explanation why patients treated with 3D-CRT more often had an LRR in the EXP group (12%) than in the STD group (6%), whereas no such difference was seen in patients treated with IMRT/VMAT (6% vs. 5%, respectively). Irradiated volumes concerning tumor coverage should not differ between the techniques and are therefore unlikely to be associated with an LRR. In addition, the excess risk of LRR in the EXP group was predominantly seen in the anastomotic region usually located centrally in the target volume. Whereas IMRT/VMAT always requires individual target volume delineation, this may not always be performed for 3D-CRT. Therefore, geographical misses may have occurred more often in the EXP than in the STD group, but more in-depth analyses are required to confirm this. Toxicity, on the other hand, may differ between the 2 RT techniques but this was not examined in this report.

An important difference between the EXP and STD groups concerns overall treatment time before surgery, which is ~40 weeks in the EXP group versus ~25 weeks in the STD group. Judging from MRI, a larger proportion of good responders were observed in the EXP group at restaging [80.1% vs. 70.1% (*P*>0.0001)], and at histopathology [93.0% vs. 87.3% (*P*=0.008)] compared with the STD group. In addition, at histopathology, a significantly higher proportion of patients had a tumor <40 mm in the EXP group (*P*=0.003), despite no difference in tumor size at baseline MRI (*P*=0.38). Although this indicates a higher response rate in the EXP group, it is conceivable that the prolonged overall treatment time may be deleterious concerning local control for the small subset of patients who are poor responders. Therefore, when a TNT regimen is used, a response evaluation should be performed during the neoadjuvant therapy and not only after the completed schedule. Although objective responses to oxaliplatin-based chemotherapy in metastatic disease are frequently seen, the chemotherapy is the weakest component of the TNT concerning cell kill capability. In addition, the observation that downsizing occurred more often among EXP patients who still had a higher rate of LRR underlines that there may be a difference between downsizing and downstaging. More low anterior resections and fewer abdominoperineal resections in the EXP group were performed despite no difference in tumor characteristics at baseline. It is conceivable that downsizing may persuade surgeons to perform less extensive surgery including more sphincter preserving procedures although microscopic tumor deposits may remain. The observation that, numerically, anastomotic recurrences occurred more often in the EXP group could support such a notion. We believe the surgical plan should be based on the baseline MRI. Moreover, we demonstrated that tumor deposits predict LRR.

ELLN, a known predictive factor for locally recurrent disease,^8,13,14^ was significantly associated with an increased risk of LRR irrespective of treatment arm. During the time of RAPIDO inclusion, the awareness of the potential importance of ELLNs and surgical proficiency for lateral lymph node dissection was less widespread than today. A lower 5-year lateral LRR rate was reported after CRT and TME with lateral lymph node dissection after the RAPIDO trial was already closed.^14^ If current guidelines^14^ regarding ELLN dissection had been applied, the LRR rate could potentially have been lower.

We classified the localization of LRR according to Kusters et al.^8^ In literature, several classification systems have been presented but most of these have not been validated against oncological outcomes.^15^ The classification by Kusters et al^8^ provides information regarding the location of the tumor but it does not distinguish whether the LRR is above and below the peritoneal reflection, which may be associated with oncological outcome.^16^ Presacral and anastomotic LRRs, axial recurrences according to the MSKCC classification system, were more often observed in the EXP group and are more often amenable to surgical treatment.^17^ This is reflected in a slightly higher rate of curatively intended surgery for the LRRs in the EXP group.

Although based on a large randomized trial, this report has several limitations. First, the RAPIDO trial was not powered for the secondary endpoint reported here. Second, a central review of MRIs, RT target volumes, dose volume histograms, delivered RT, and histopathology specimens have not yet been performed, and information was mostly retrieved by CRFs. However, MRIs and histopathological specimens are currently being revised. Third, restaging MRI was performed in most patients, but not in all. In addition, there may be unrecorded tumor characteristics, and perioperative or intraoperative variables not accounted for.

The outcomes previously reported from the RAPIDO trial showed important gains from a TNT approach including a significant decrease in DrTF at 3 years and a doubled rate of pCR.^4^ These gains were achieved with comparable health-related quality of life, bowel function, and late toxicity at 3 years.^18^ However, the results after an R0/R1 surgery reported herein, showing statistically significantly decreased locoregional control rates in the EXP group prompt further refinements of the TNT approach. Early response assessment with interruption of the weakest part of the treatment, i.e., the chemotherapy, in case no response or even progression is seen, adequate coverage of the tumor cell containing tissue volumes, dose escalation, increased rate of lateral lymph node dissection on indication, and a surgical plan based on initial pretreatment MRI may all be important.

## Supplementary Material
