# Supplementary material for: Locoregional Failure During and After Short-course Radiotherapy Followed by Chemotherapy and Surgery Compared With Long-course Chemoradiotherapy and Surgery: A 5-Year Follow-up of the RAPIDO Trial
Source: Ann Surg. 2023 Jan 20;278(4):e766–72. doi: 10.1097/SLA.0000000000005799 (PMC10481913; doi:10.1097/SLA.0000000000005799)
Supplement: Supplementary file 1 [file sla-278-e766-s001.docx]

Online supplement

Table of Contents

|  |  | Page |
| --- | --- | --- |
| Table S1 | Baseline characteristics and radiation techniques of all eligible patients and of patients who developed a locoregional failure according to randomization | 2 |
| Table S2 | Characteristics of patients with early locoregional failure | 4 |
| Table S3 | Univariate and multivariate Cox regression analyses of clinical characteristics regarding patients who **did not** undergo a curative resection. | 5 |
| Table S4 | High-risk criteria, radiation, surgical and pathological characteristics of patients who developed a locoregional recurrence | 6 |
| Table S5 | Location of the locoregional recurrences (LRR) | 8 |
| Figure S2 | Overall survival after diagnosis of a locoregional failure. | 9 |
|  | Definition of the location of LRR | 10 |

| **Table S1**  Baseline characteristics and radiation techniques of all eligible patients and of patients who developed a locoregional failure according to randomization | | included in the  **locoregional failure** analyses. | | | | | |  | in whom a  **locoregional failure** was detected | | | | |
| --- | --- | --- | --- | --- | --- | --- | --- | --- | --- | --- | --- | --- | --- |
|  | | Experimental  (n=460) | | | Standard-care  (n=446) | | *P*-value |  | Experimental  (n=54) | | Standard-care  (n=36) | | *P*-value |
| Gender | | | | | | | 0.17 |  |  |  |  |  | 0.63 |
| Male  Female | 299  161 | | | (65.0)  (35.0) | 309  137 | (69.3)  (30.7) |  |  | 38  16 | (70)  (30) | 27  9 | (75)  (25) |  |
| Age (years) | | | | | | | 0.72 |  |  |  |  |  | 0.83 |
| Median (IQR) | 62 | | | (55-68) | 62 | (55-68) |  |  | 61 | (55-67) | 61 | (54-65) |  |
| ECOG |  | | |  |  |  | 0.60 |  |  |  |  |  | 0.46 |
| 0  1 | 368  92 | | | (80.0)  (20.0) | 363  83 | (81.4)  (18.6) |  |  | 45  9 | (83)  (17) | 32  4 | (89)  (11) |  |
| High-risk criteria† | | | | | | |  |  |  |  |  |  |  |
| cT4  cN2  Enlarged lateral nodes  EMVI +  MRF + | 149  317  70  165  309 | | | (32.4)  (68.9)  (15.2)  (35.9)  (67.2) | 138  310  73  150  311 | (30.9)  (69.5)  (16.4)  (33.6)  (69.7) | 0.64  0.85  0.64  0.48  0.41 |  | 17  41  13  19  39 | (32)  (76)  (24)  (35)  (72) | 13  29  10  19  30 | (36)  (81)  (28)  (53)  (83) | 0.65  0.61  0.69  0.10  0.22 |
| Distance from anal verge on endoscopy | | | | | | | 0.24§ |  |  |  |  |  | 0.85§ |
| < 5 cm  5 – 10 cm  ≥ 10 cm  Unknown | 101  181  146  32 | | | (22.0)  (39.3)  (31.7)  (7.0) | 113  153  149  31 | (25.3)  (34.3)  (33.4)  (7.0) |  |  | 9  20  19  6 | (17)  (37)  (35)  (11) | 8  14  12  2 | (22)  (39)  (33)  (6) |  |
| Radiation technique | | | | | | | 0.11§ |  |  |  |  |  | **0.029** |
| 3D-CRT  IMRT/VMAT  Unknown | | | 339  121  - | (73.7)  (26.3) | 306  139  1 | (68.6)  (31.2)  (0.2) |  |  | 45  9 | (83)  (17) | 22  13 | (63)  (37) |  |
| Type of locoregional failure | | |  |  |  |  |  |  |  |  |  |  | 0.28 |
| Early locoregional failure*  LRR after no surgery**  LRR after R0 resection  LRR after R1 resection | | |  |  |  |  |  |  | 10  -  28  16 | (19)  (52)  (30) | 10  2  15  9 | (28)  (6)  (42)  (25) |  |
| Distant metastases | | |  |  |  |  |  |  |  |  |  |  | 0.45 |
| Yes, before/simultaneously with LRF  Yes, after LRF  No | | |  |  |  |  |  |  | 30  9  15 | (56)  (17)  (28) | 24  6  6 | (67)  (17)  (17) |  |
| Data is presented as n (%). Percentages may not equal to 100 due to rounding.  *EMVI* extramural vascular invasion; *MRF* mesorectal fascia; *3D-CRT* three-dimensional conformal radiation therapy; *IMRT*  intensity-modulated radiation therapy; *VMAT* volumetric-modulated arc therapy; *LRR* locoregional recurrence.  † MRI defined.  § p-value calculated in patients in which the result was known.  * Early locoregional failure was defined as no resection surgery for other reasons than entering a W&W strategy or an R2 resection.  **Patients who were categorized as having ‘refused surgery’ were grouped together with those entering a W&W strategy since it turned out that they did not have any remaining tumor. A W&W strategy was not according to the protocol; several physicians then rather wrote that the patient ‘refused surgery’ than that they entered that strategy. | | | | | | | | | | | | | |

| **Table S2**  Characteristics of patients with early locoregional failure | | | | |
| --- | --- | --- | --- | --- |
|  | Experimental  (n=10) | | Standard-care  (n=10) | |
| Gender | | | | |
| Male  Female | 7  3 |  | 8  2 |  |
| Age (years) | | | | |
| Median (range) | 60 (56-66) |  | 58 (51-63) |  |
| High-risk criteria † | | | | |
| cT4  cN2  Enlarged lateral nodes  EMVI +  MRF + | 3  8  4  5  9 |  | 6  9  3  6  9 |  |
| Distance from anal verge on endoscopy | | | | |
| < 5 cm  5 – 10 cm  ≥ 10 cm  Unknown | 1  4  3  2 |  | 2  2  6  - |  |
| Compliance to neoadjuvant treatment |  |  |  |  |
| All RT fractions  ≥75% of prescribed preoperative  chemotherapy  At least 45 Gy | 10  8  - |  | -  -  8 |  |
| Distant metastases |  |  |  |  |
| Yes; before/synchronously with LRF  Yes; after LRF  No | 8  -  2 |  | 10  -  - |  |
| Data is presented as n.  *EMVI* extramural vascular invasion; *MRF* mesorectal fascia; *RT* radiotherapy; *LRF* locoregional failure.  † MRI defined | | | | |

| **Table S3**  Univariate and multivariate Cox regression analyses of clinical characteristics regarding patients who **did not** undergo a curative resection* | | | | | | | | | | |
| --- | --- | --- | --- | --- | --- | --- | --- | --- | --- | --- |
| Variable | Category | Univariate analyses | | | |  | Multivariate analyses | | | |
|  |  | n | HR (95% CI) | | *P*-value |  | n | HR (95% CI) | | *P*-value |
| Treatment | Standard-care | 20 | 1 | |  |  |  |  |  |  |
|  | Experimental | 29 | 0.61 (0.22-1.75) | | 0.361 |  |  |  | |  |
|  |  |  |  | |  |  |  |  |  |  |
| Distance from anal verge (endoscopy) † | < 5 cm | **18** | **1** | | **0.049** |  | 18 | 1 |  | 0.120 |
|  | 5-10 cm | **16** | **6.01 (0.70-51.51)** | |  |  | 16 | 5.43 (0.63-46.90) | |  |
|  | ≥ 10 cm | **13** | **12.95 (1.55-108.30)** | |  |  | 13 | 9.88 (1.11-88.11) | |  |
|  |  |  |  |  |  |  |  |  |  |  |
| Clinical T4 | No | 37 | 1 | |  |  |  |  |  |  |
|  | Yes | 12 | 1.34 (0.42-4.27) | | 0.586 |  |  |  |  |  |
|  |  |  |  |  |  |  |  |  |  |  |
| Clinical N2 | No | 12 | 1 | |  |  |  |  |  |  |
|  | Yes | 37 | 2.22 (0.50-9.96) | | 0.296 |  |  |  |  |  |
|  |  |  |  |  |  |  |  |  |  |  |
| Enlarged lateral lymph nodes | No | **40** | **1** | |  |  | 40 | 1 | |  |
|  | Yes | **9** | **5.44 (1.87-15.87)** | | **0.002** |  | 7 | 3.20 (0.85-12.00) | | 0.084 |
|  |  |  |  |  |  |  |  |  |  |  |
| EMVI+ | No | **34** | **1** | |  |  | 33 | 1 | |  |
|  | Yes | **15** | **3.82 (1.32-11.05)** | | **0.014** |  | 14 | 2.00 (0.52-7.66) | | 0.312 |
|  |  |  |  |  |  |  |  |  |  |  |
| MRF+ | No | 15 | 1 | |  |  |  |  | |  |
|  | Yes | 34 | 3.07 (0.69-13.74) | | 0.142 |  |  |  | |  |
| * No curative resection entails no resection for any reason or an R2 resection  † In 2 patients (who developed failure up until curative surgery), the distance from the anal verge was unknown, these patients were set to missing. Therefore, the number of patients included in the multivariate analyses is lower.  *EMVI* extramural vascular invasion; *MRF* mesorectal fascia | | | | | | | | | | |

| **Table S4**  High-risk criteria, radiation, surgical and pathological characteristics of patients who developed a locoregional recurrence | | | | | |
| --- | --- | --- | --- | --- | --- |
|  | Experimental  (n=44) | | Standard-care (n=26) | | *P*-value |
| High-risk criteria at baseline† |  |  |  |  |  |
| cT4  cN2  Enlarged lateral nodes  EMVI +  MRF + | 14  33  9  14  30 | (32)  (75)  (21)  (32)  (68) | 7  20  7  13  21 | (27)  (77)  (27)  (50)  (81) | 0.66  0.86  0.53  0.13  0.25 |
| Radiation technique |  |  |  |  | 0.14 |
| 3D-CRT  IMRT/VMAT | 37  7 | (84)  (16) | 18  8 | (69)  (31) |  |
| Type of resection |  |  |  |  | 0.18 |
| Anterior resection, PME  Low anterior resection, TME  Abdominoperineal resection  Hartmann’s procedure  Other  Refused surgery | -  23  12  8  1  - | (52)  (27)  (18)  (2) | -  9  11  3  1  2 | (35)  (42)  (12)  (4)  (8) |  |
| Resection status (distance to distal margin, according to Wittekind) |  |  |  |  | 0.93§ |
| R0 > 1 mm  R1 ≤ 1 mm  Refused surgery | 28  16 | (64)  (36) | 15  9  2 | (58)  (35)  (8) |  |
| Pathological complete response |  |  |  |  | 0.19§ |
| No  Yes  Unknown | 40  3  1 | (91)  (7)  (2) | 23  -  3 | (88)  (12) |  |
| Mesorectum |  |  |  |  |  |
| Intact  Breached  Missing | 29  9  6 | (66)  (21)  (14) | 22  1  3 | (85)  (4)  (12) | **0.048§** |
| Differentiation grade |  |  |  |  | 0.92§ |
| Well  Moderate  Poor  Not assessed/unknown | 9  22  8  5 | (21)  (50)  (18)  (11) | 7  9  6  2 | (27)  (35)  (23)  (8) |  |
| Pathological T-stage |  |  |  |  | 0.38§ |
| ypT0  ypTis  ypT1  ypT2  ypT3  ypT4  Unknown/refused surgery | 3  -  -  5  30  6  - | (7)  (11)  (68)  (14) | -  -  -  4  16  4  2 | (15)  (62)  (15)  (8) |  |
| Pathological N-stage |  |  |  |  | 0.26§ |
| ypN0  ypN1  ypN2  Unknown/refused surgery | 21  14  9  - | (48)  (32)  (21) | 9  7  8  2 | (35)  (27)  (31)  (8) |  |
| Distance to circumferential resection margin of the tumor |  |  |  |  | 0.67§ |
| CRM- (>1 mm)  CRM+ (≤1 mm)  Unknown/refused surgery | 28  16 | (64)  (36) | 14  10  2 | (54)  (39)  (8) |  |
| Tumor size at baseline MRI |  |  |  |  | 0.44§ |
| <40mm  ≥40mm  Unknown | 4  38  2 | (9)  (86)  (5) | 4  20  1 | (15)  (78)  (4) |  |
| Tumor size at histopathology |  |  |  |  | 0.12§ |
| <40mm  ≥40mm  Unknown/refused surgery | 33  10  1 | (75)  (23)  (2) | 14  10  2 | (54)  (38)  (8) |  |
| Data is presented as locoregional recurrence/population in numbers and percentages. Percentages may not equal 100% due to rounding  *LRR* Locoregional recurrence; *EMVI* extramural vascular invasion; *MRF* mesorectal fascia; *IMRT* intensity-modulated radiation therapy; *VMAT* volumetric-modulated arc therapy; *CRM* circumferential resection margin.  † MRI defined  § p-value calculated in patients in which the value was known.  * Distance was missing in 4 patients of the experimental group and in 1 patient of the standard-care group. | | | | | |

| **Table S5**  Location of the locoregional recurrences (LRR) | | |  |
| --- | --- | --- | --- |
|  | Experimental  (n=450) | Standard-care  (n=436) | |
| No LRR† | 406 | 410 | |
| All locations of LRR | N=44 | N=26 | |
| Lateral | 8 | 7 | |
| Presacral | 19 | 9 | |
| Anterior | 11 | 9 | |
| Anastomosis | 14 | 3 | |
| Perineal | 5 | 3 | |
| Other location | - | 3 | |
| Single location of LRR | N=32 | N=18 | |
| Lateral | 4 | 3 | |
| Presacral | 12 | 3 | |
| Anterior | 7 | 5 | |
| Anastomosis | 7 | 2 | |
| Perineal | 2 | 2 | |
| Other location | - | 3 | |
| Multifocal locations LRR | N=12 | N=8 | |
| Lateral & anterior | 1 | 2 | |
| Lateral & presacral | 2 | 1 | |
| Lateral & perineal | 1 | - | |
| Presacral & anterior | 1 | 2 | |
| Presacral & anastomosis | 3 | 1 | |
| Presacral & perineal | - | 1 | |
| Anastomosis & perineal | 2 | - | |
| Anastomosis & anterior | 1 | - | |
| Lateral, presacral & anterior | - | 1 | |
| Presacral, anterior & anastomosis | 1 | - | |
| Data is presented as n.  *LRR* Locoregional recurrence  † Consists of no LRF or early failure. | | |  |

| 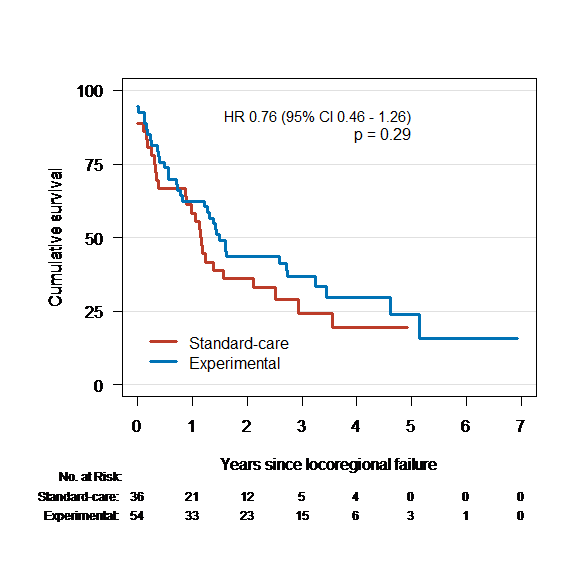 |
| --- |
| **Figure S1**  Overall survival after diagnosis of a locoregional failure.  The numbers are actual numbers. |

**Definition of the location of LRR**

The location of recurrent disease was recorded in the CRFs and centrally reviewed by imaging reports (MRI, CT, PET) and/or histology reports. Locations were classified according to Kusters *et al.* (1):

- Lateral: pelvic side wall, immediately behind posterior ischiac spine, in the obturator compartment, or along iliac vessels;
- Presacral: predominantly midline, in contact with sacral bone;
- Anterior: predominantly midline, involving bladder, uterus, vagina, seminal vesicles, or prostate;
- Anastomosis: after low anterior resection or low Hartmann, at the staple line;
- Perineal: perineum, anal sphincter complex with surrounding perianal and ischiorectal space;
- Other.

1: Kusters M, Marijnen CAM, van de Velde CJH, Rutten HJT, Lahaye MJ, Kim JH, et al. Patterns of local recurrence in rectal cancer; a study of the Dutch TME trial. European Journal of Surgical Oncology. 2010;36(5):470–6.
